# Supplementary figures and images for: Blockade of interleukin-6 (IL-6) signaling in dedifferentiated liposarcoma (DDLPS) decreases mouse double minute 2 (MDM2) oncogenicity via alternative splicing
Source: PLoS One. 2025 Sep 17;20(9):e0299962. doi: 10.1371/journal.pone.0299962 (PMC12443315; doi:10.1371/journal.pone.0299962)

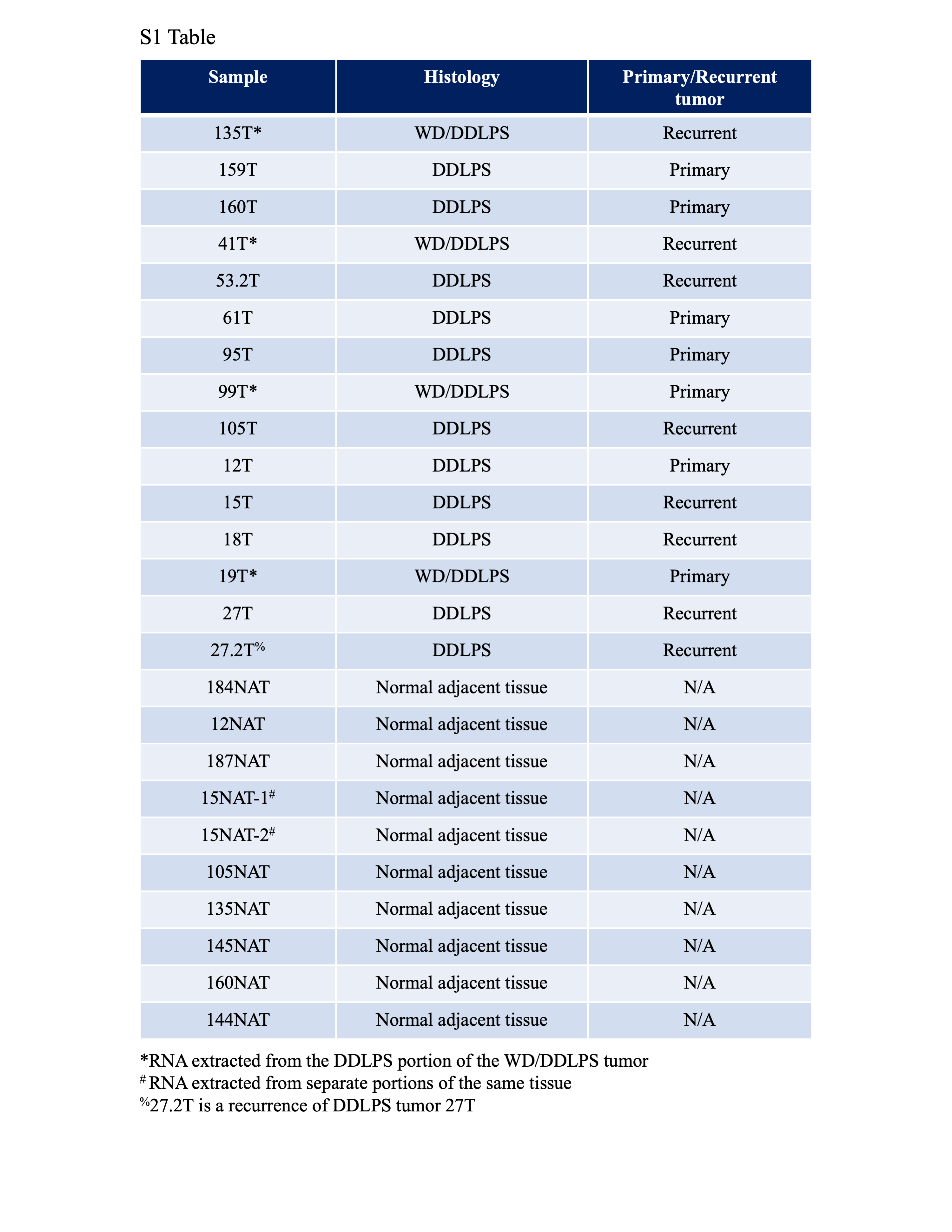

Supplement: S1 Table — (TIFF) [file pone.0299962.s001.tiff]

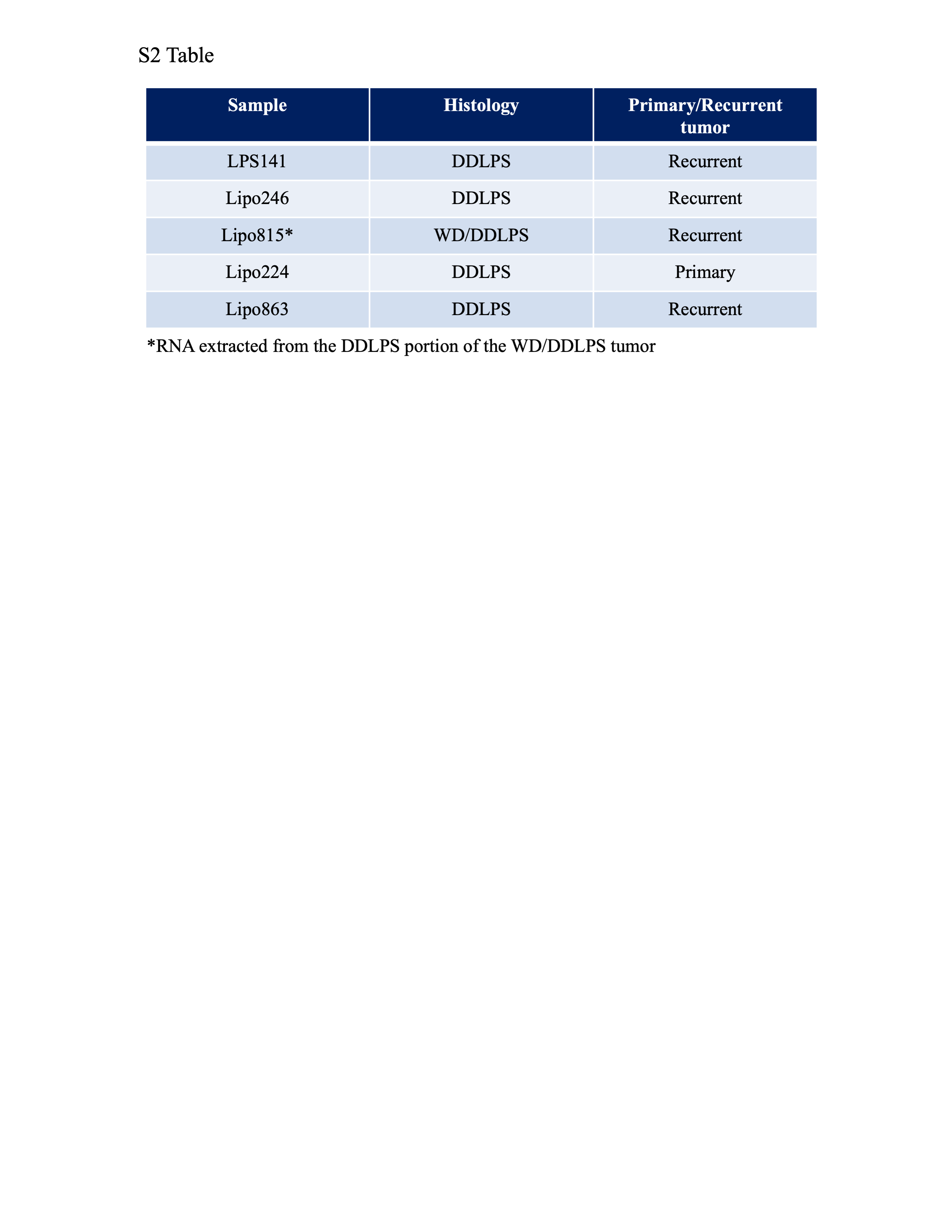

Supplement: S2 Table — (TIFF) [file pone.0299962.s002.tiff]

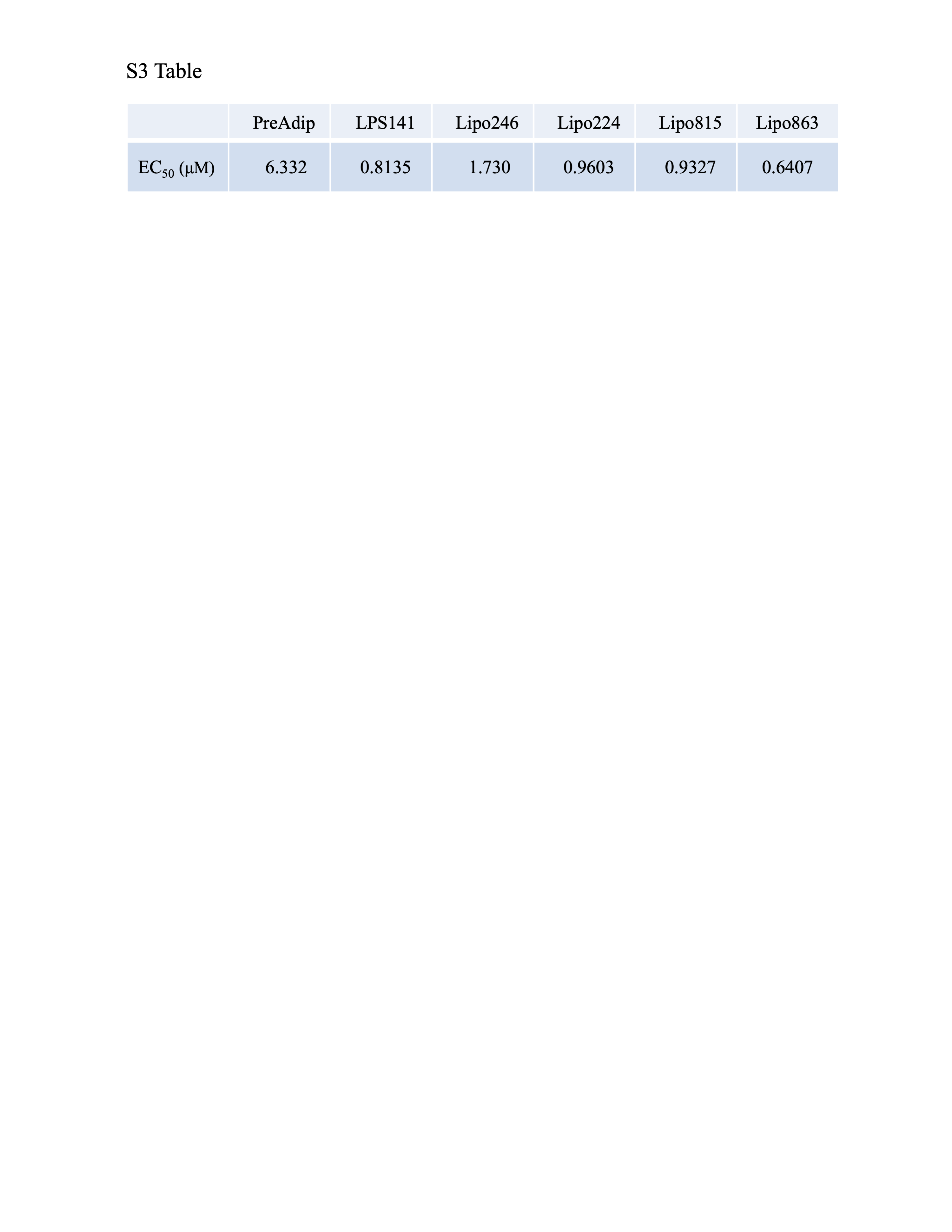

Supplement: S3 Table — (TIFF) [file pone.0299962.s003.tiff]

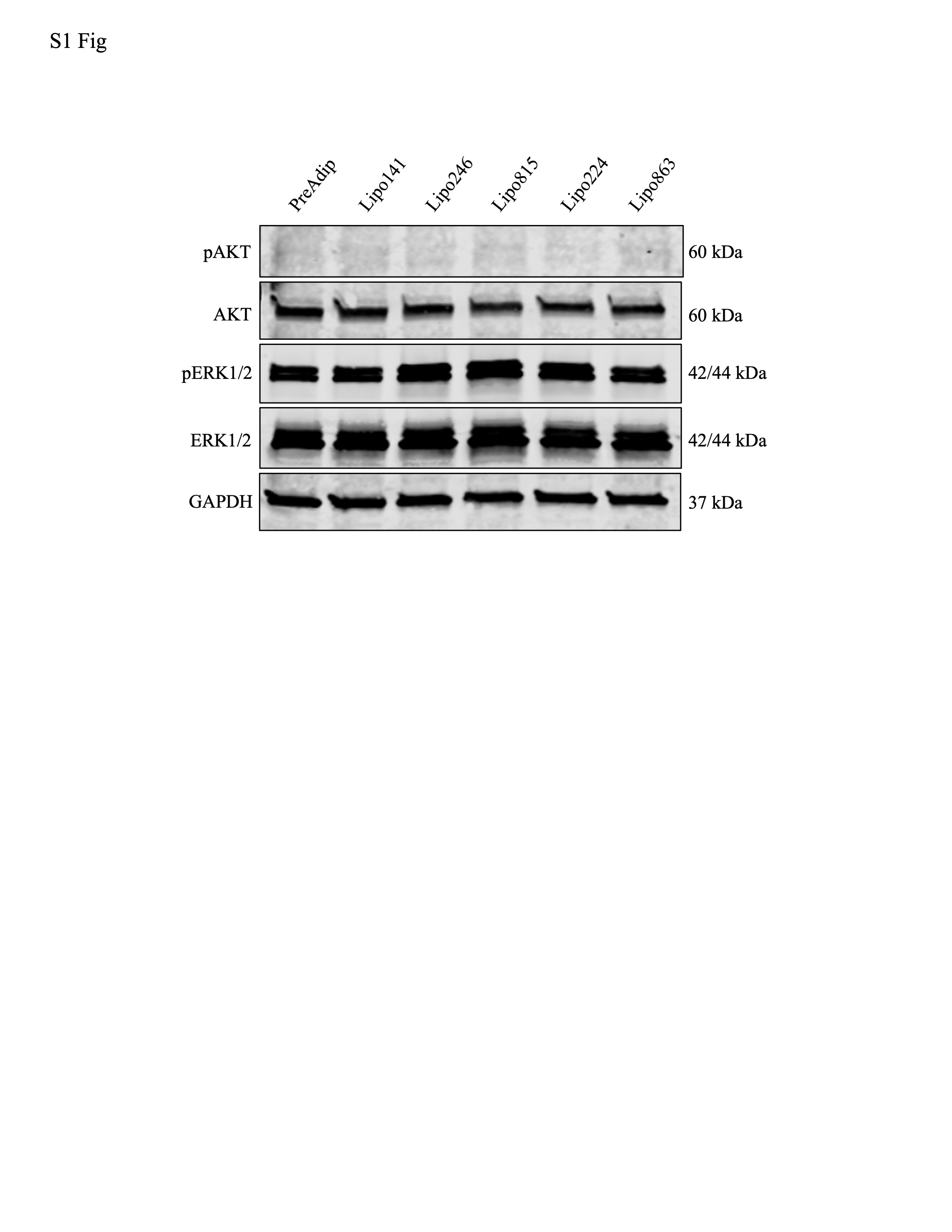

Supplement: S1 Fig — AKT and ERK1/2 protein expression in serum-starved DDLPS cells (24h). (TIFF) [file pone.0299962.s004.tiff]

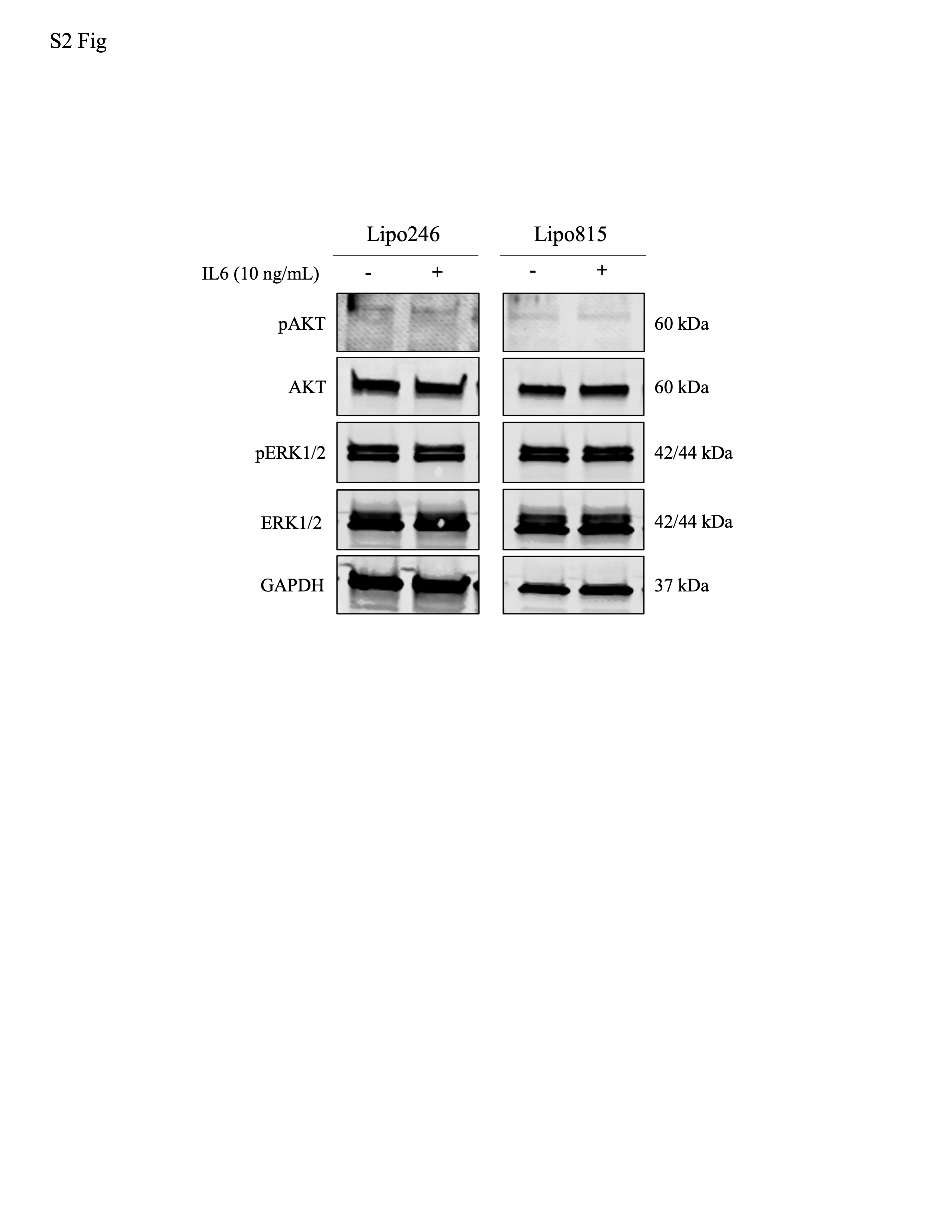

Supplement: S2 Fig — AKT and ERK1/2 protein expression in Lipo246 and Lipo815 cells after treatment with IL6 (10 ng/mL, 20 min). (TIFF) [file pone.0299962.s005.tiff]

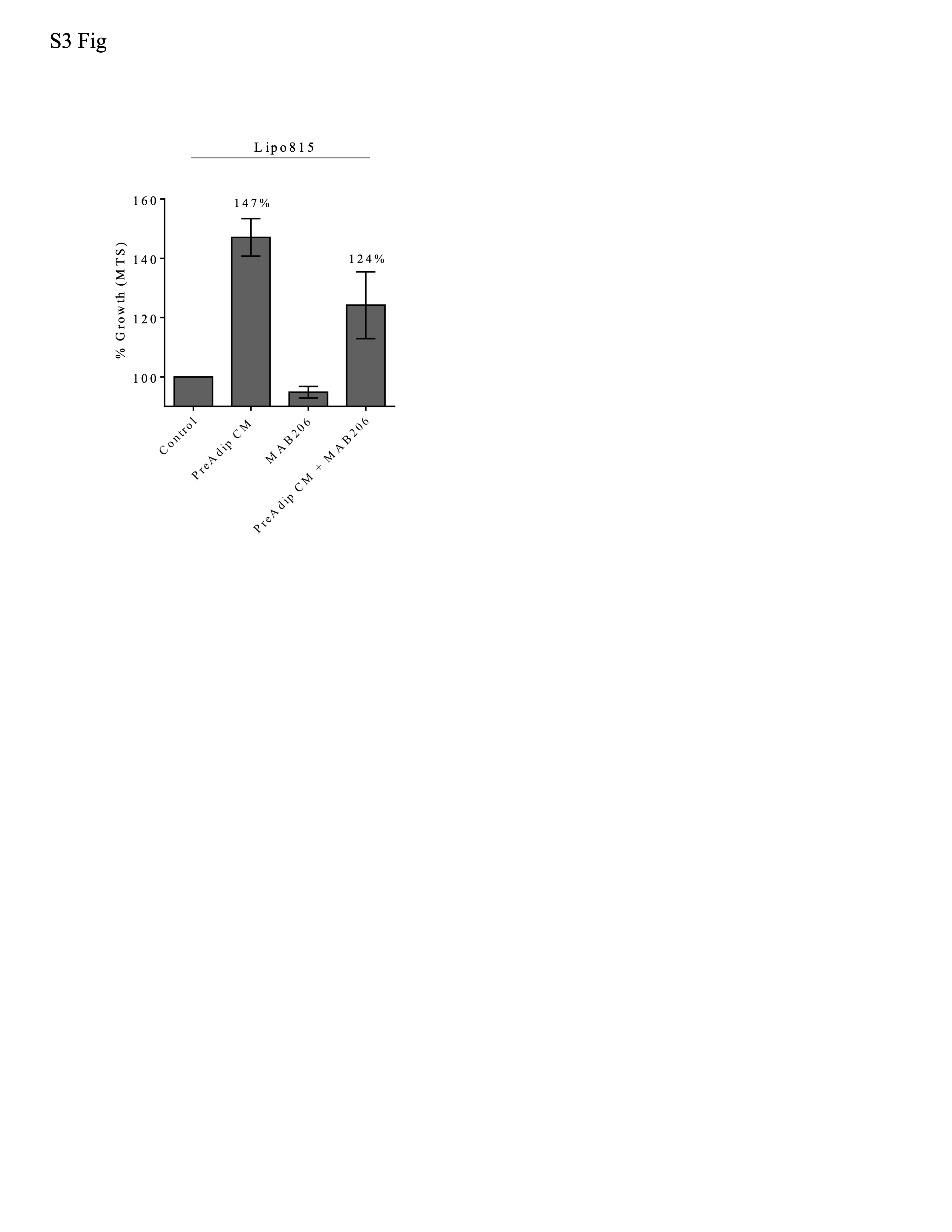

Supplement: S3 Fig — MTS evaluation of Lipo815 cells cultured in the absence or presence of PreAdip-derived CM and MAB206 (0.6 μg/mL) for 96h. (TIFF) [file pone.0299962.s006.tiff]

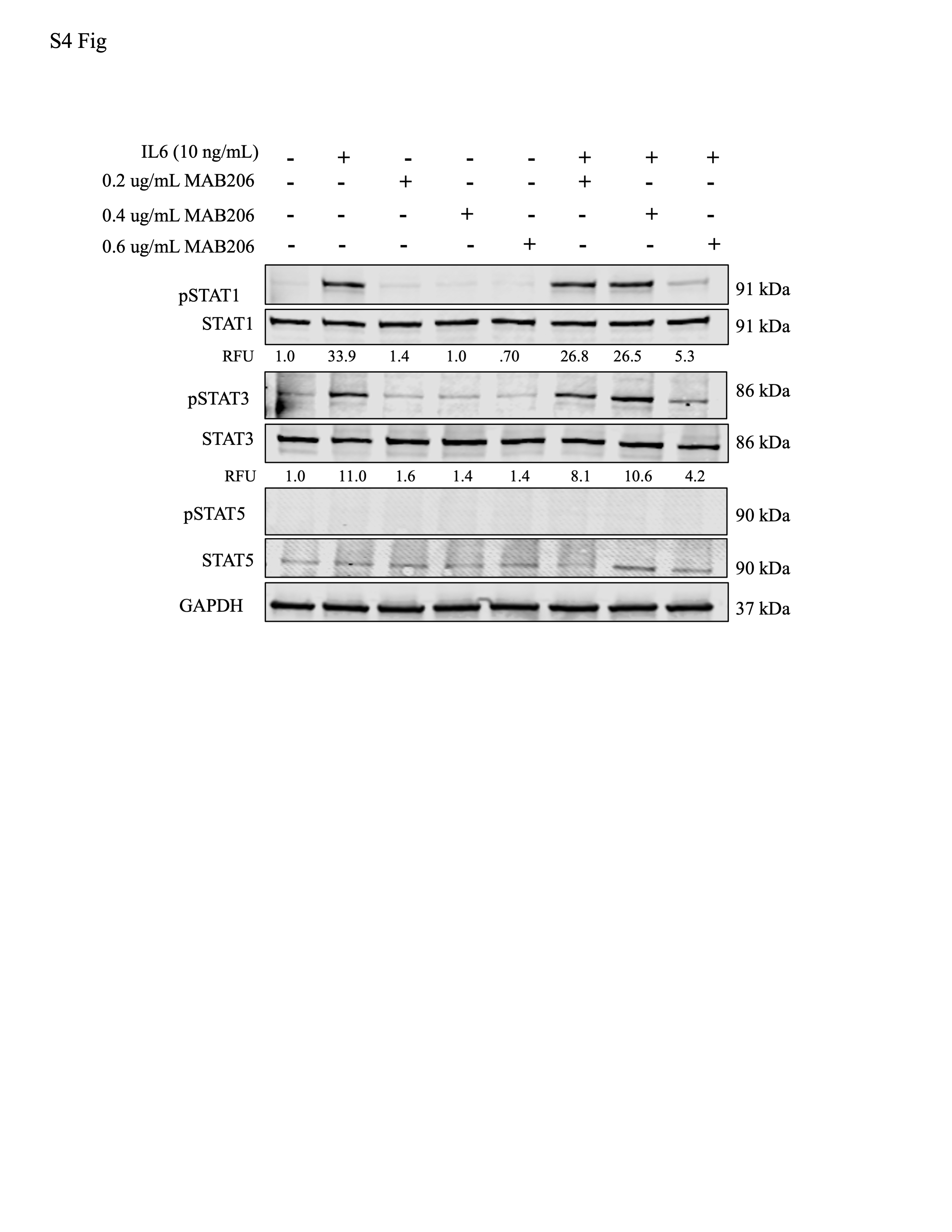

Supplement: S4 Fig — STAT1, STAT3 and STAT5 protein expression in Lipo815 cells pretreated with MAB206 (0.6 μg/mL, 1h) prior to the addition of IL6 (10 ng/mL, 20 min). (TIFF) [file pone.0299962.s007.tiff]

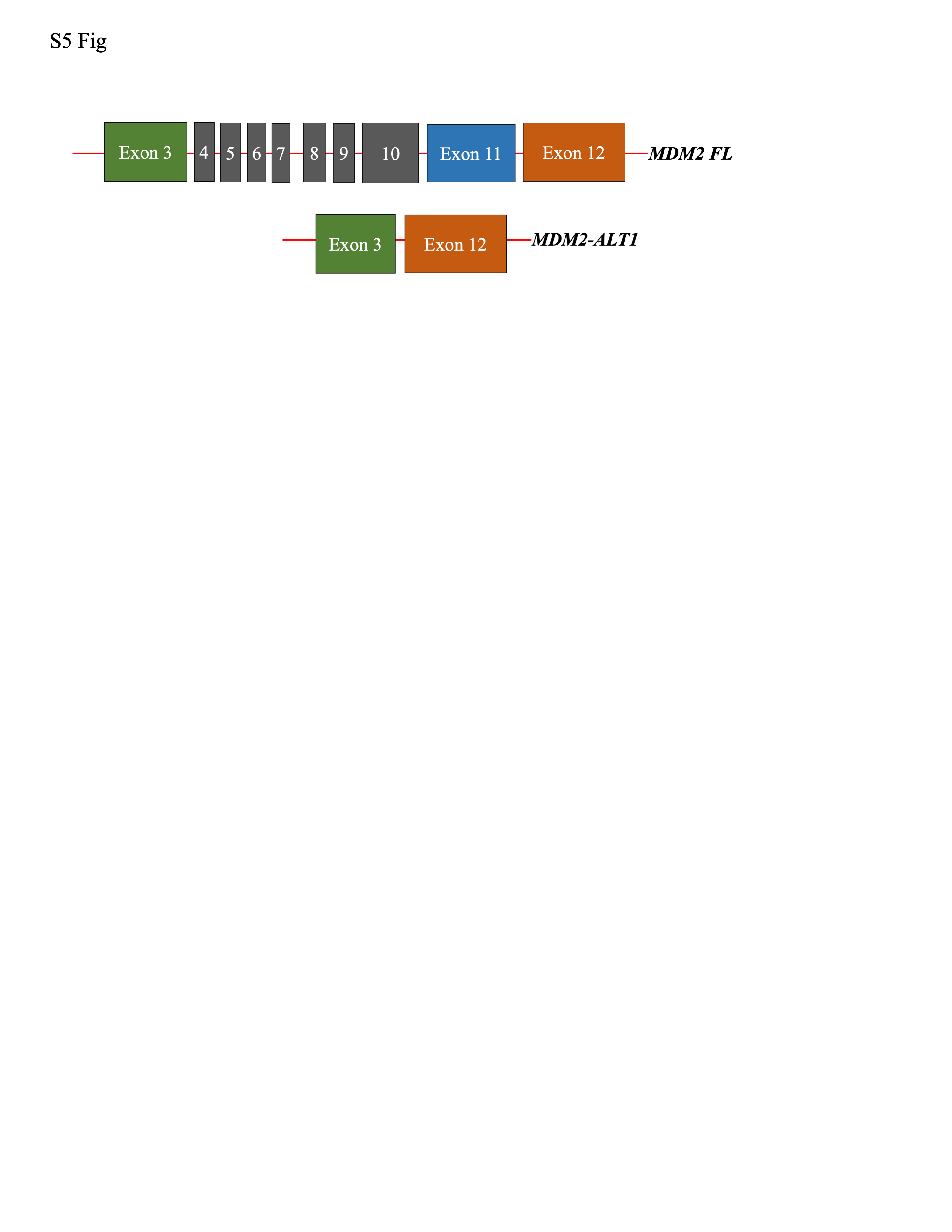

Supplement: S5 Fig — (TIFF) [file pone.0299962.s008.tiff]

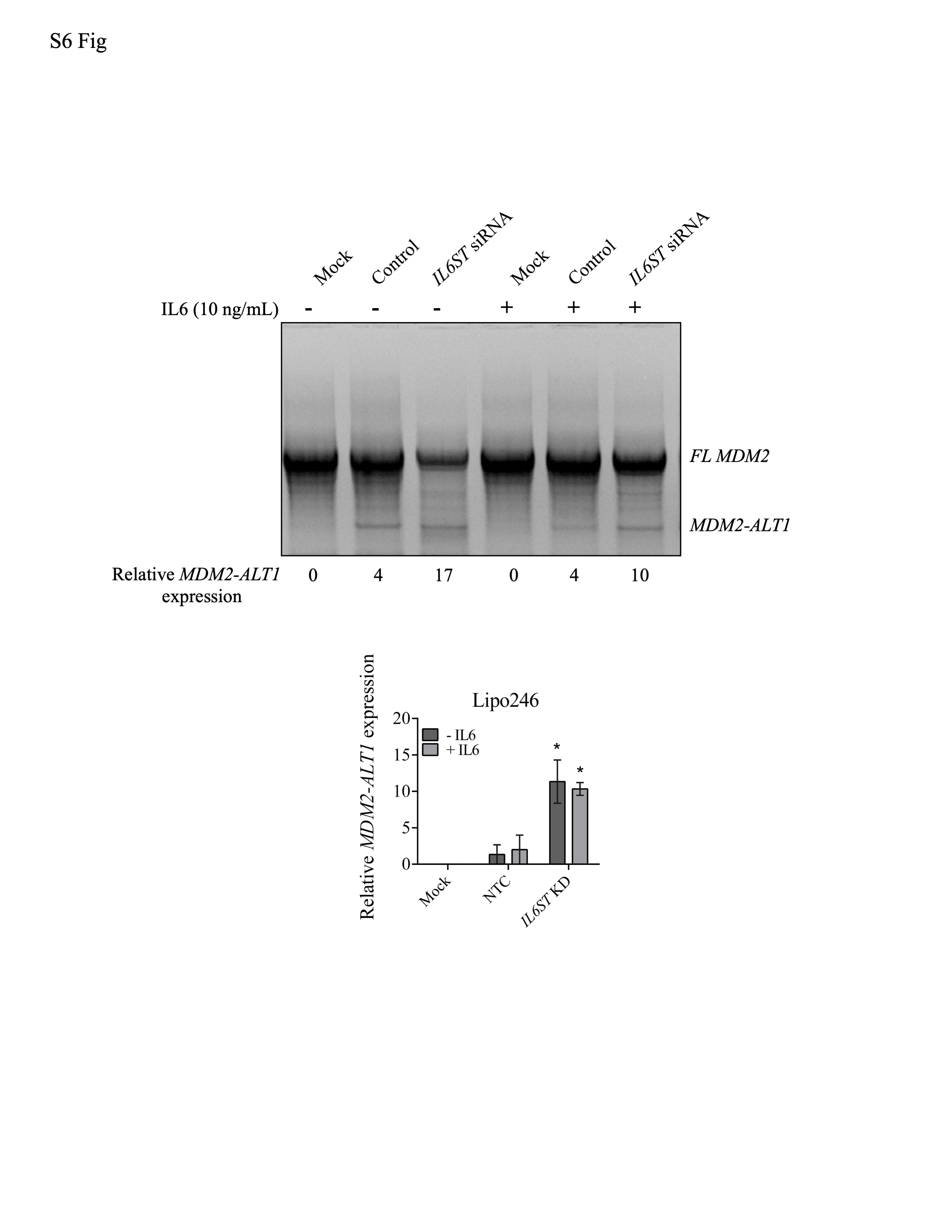

Supplement: S6 Fig — (TIFF) [file pone.0299962.s009.tiff]
