# Supplementary material for: Blockade of interleukin-6 (IL-6) signaling in dedifferentiated liposarcoma (DDLPS) decreases mouse double minute 2 (MDM2) oncogenicity via alternative splicing
Source: PLoS One. 2025 Sep 17;20(9):e0299962. doi: 10.1371/journal.pone.0299962 (PMC12443315; doi:10.1371/journal.pone.0299962)
Supplement: S1 Raw Images — (PDF) [file pone.0299962.s010.pdf]

Fig 1D. Baseline GP130 protein expression in Preadipocyte and DDLPS cells

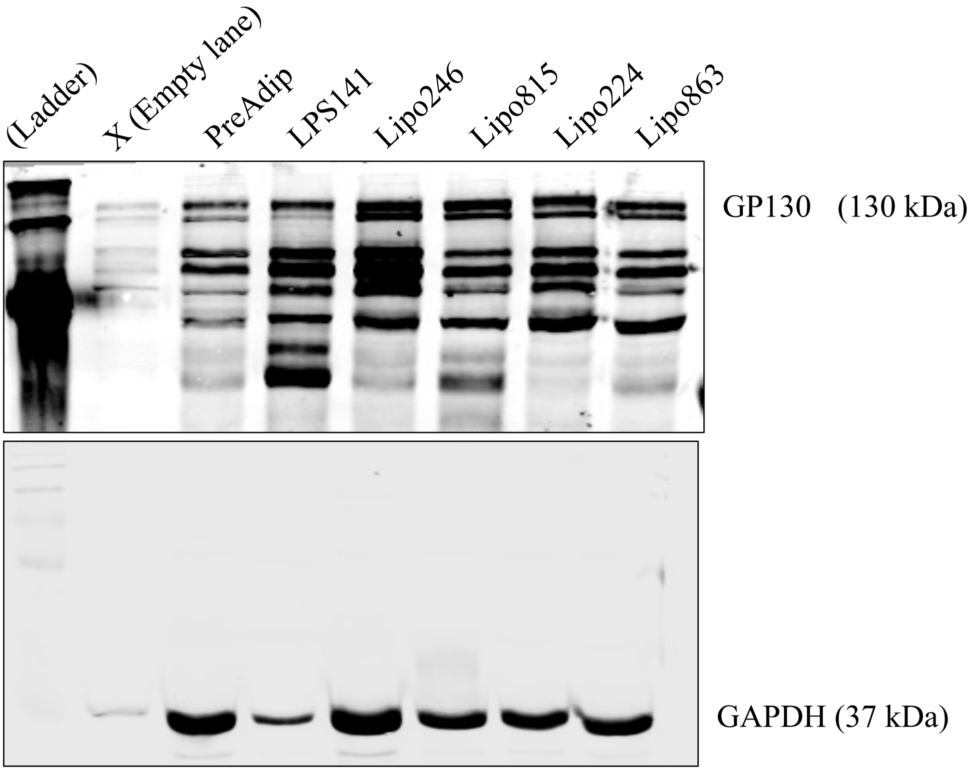

Imaging and protein expression analyses were performed with the Odyssey CLx imager.

Fig 1E. IL6-mediated STAT1 and STAT3 Activation in DDLPS cells

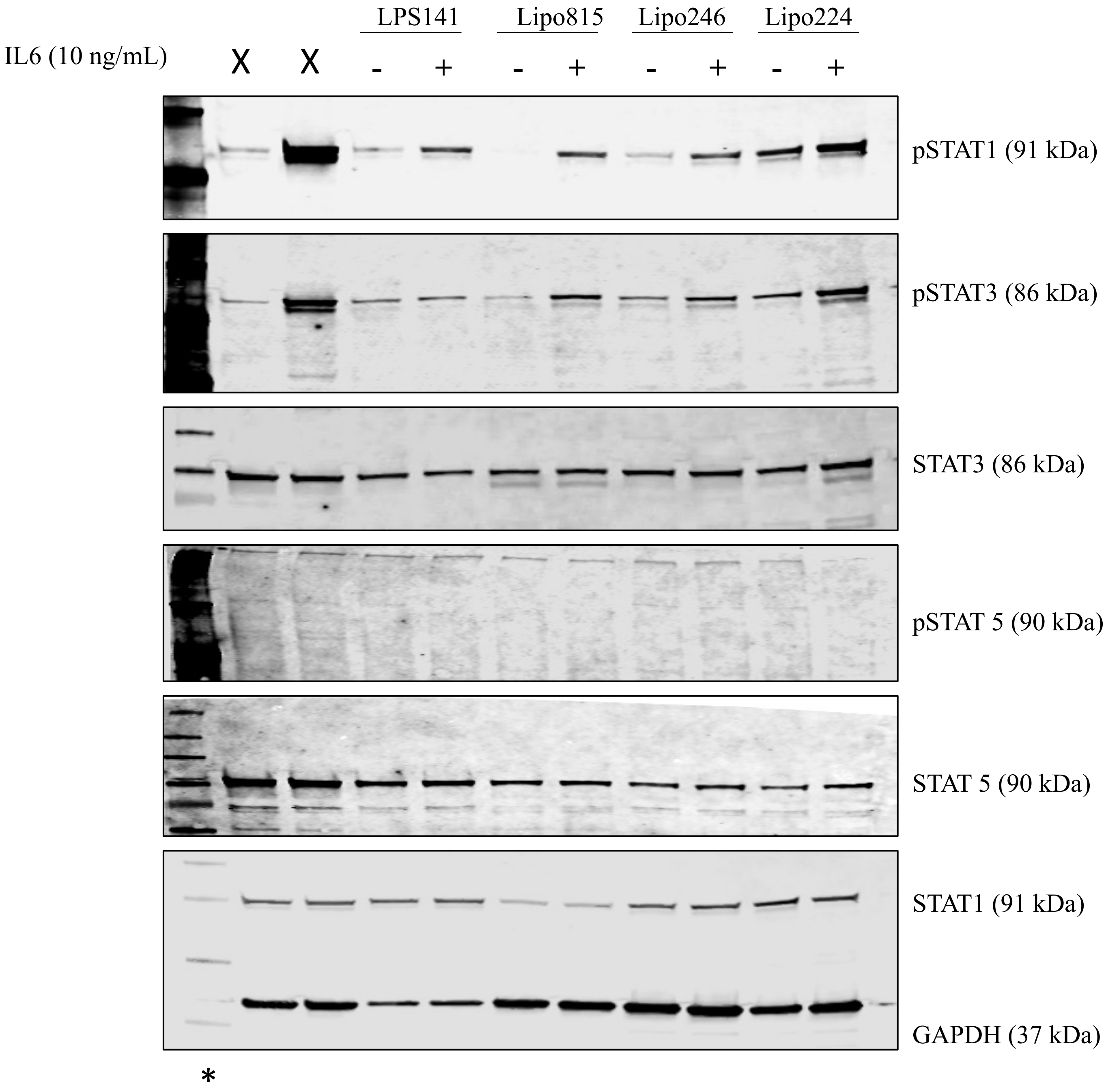

* First lane denotes ladder

Imaging and protein expression analyses were performed with the Odyssey CLx imager.

Fig 1F. Loss of STAT1 and STAT3 Activation in Lipo815 cells after GP130 Receptor Knockdown

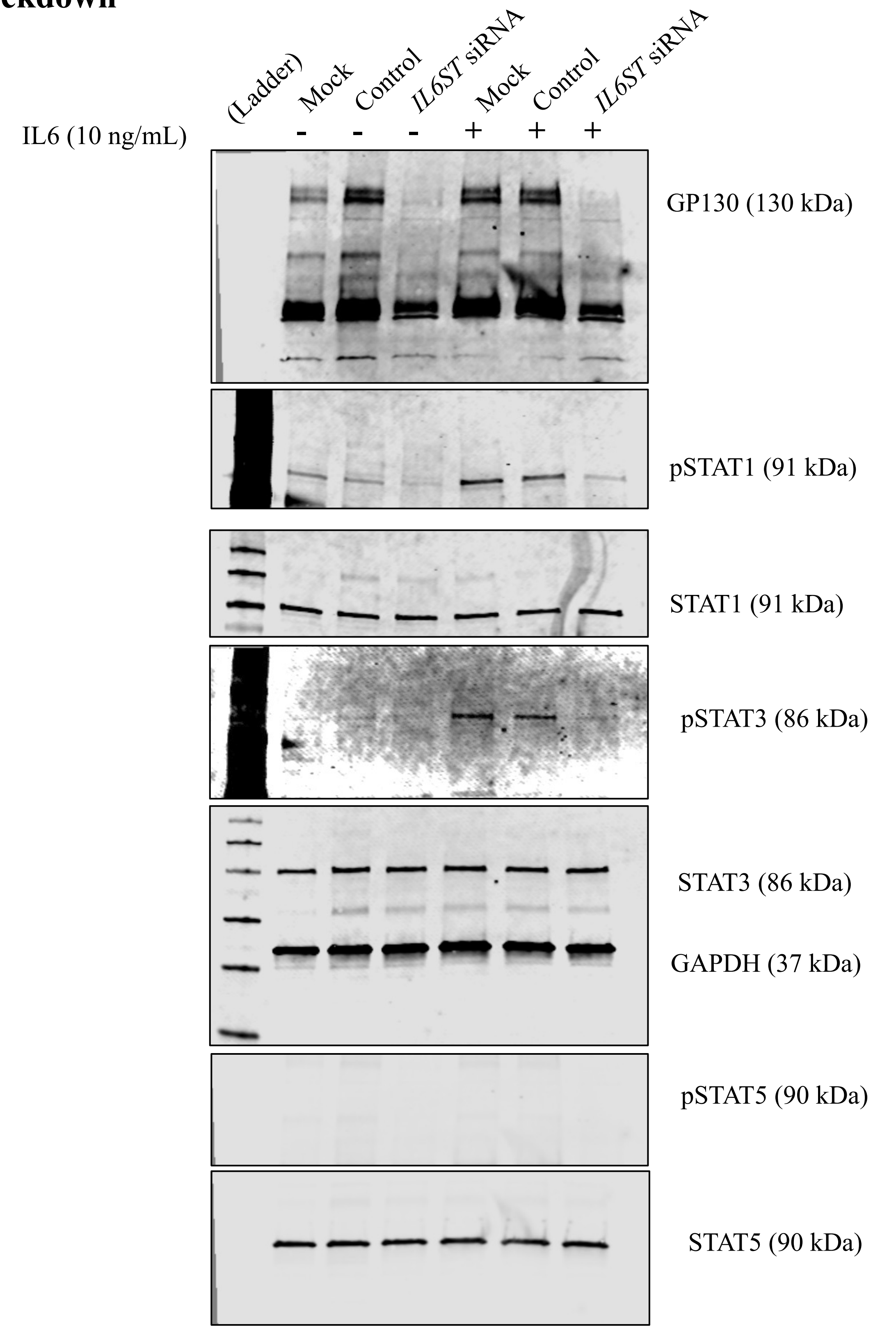

Imaging and protein expression analyses were performed with the Odyssey CLx imager.

Fig 4A. GP130 Activation Increases DDLPS MDM2 Levels

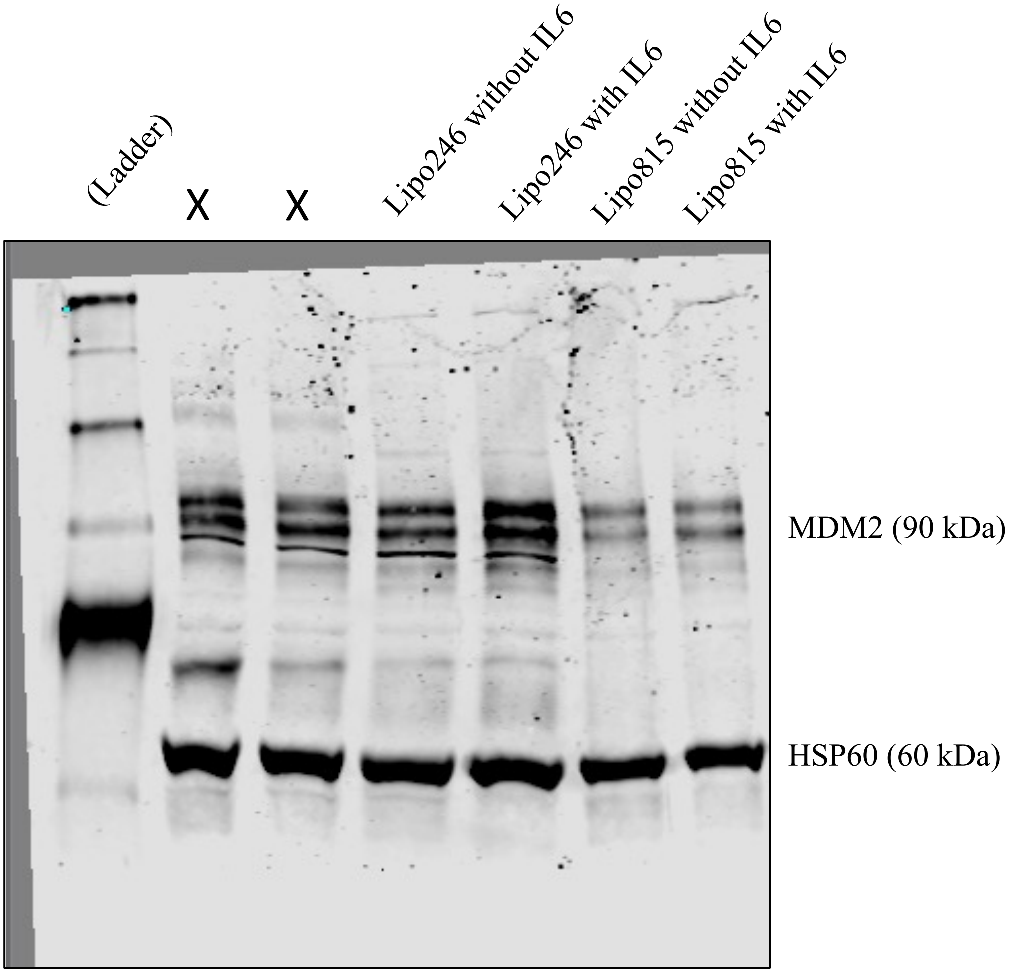

Imaging and protein expression analyses were performed with the Odyssey CLx imager.

Fig 4B. GP130 Knockdown Decreases Lipo815 MDM2 Levels

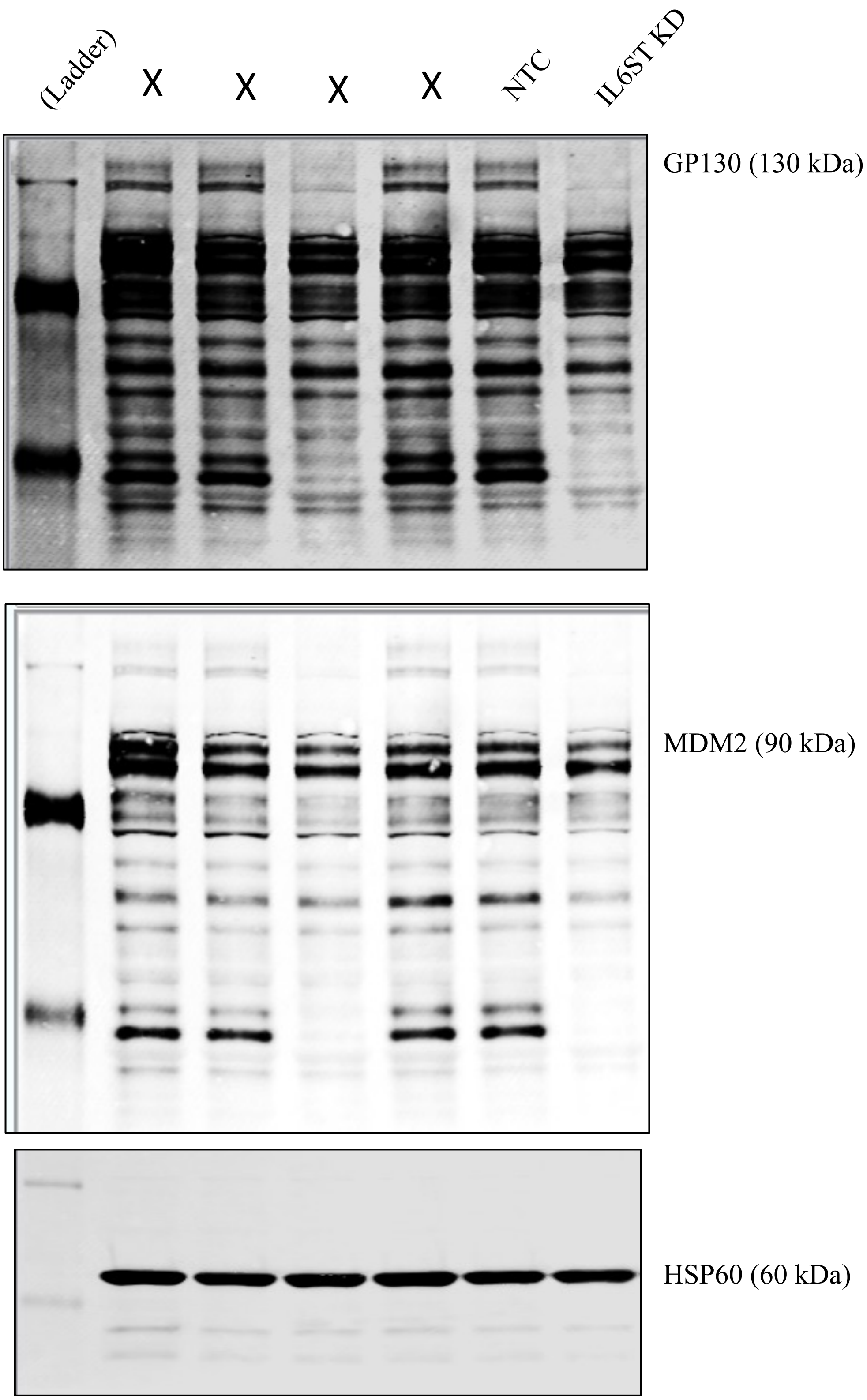

Imaging and protein expression analyses were performed with the Odyssey CLx imager.

Fig 7A. GP130 protein expression in Lipo246 and Lipo815 cells after SC144 treatment.

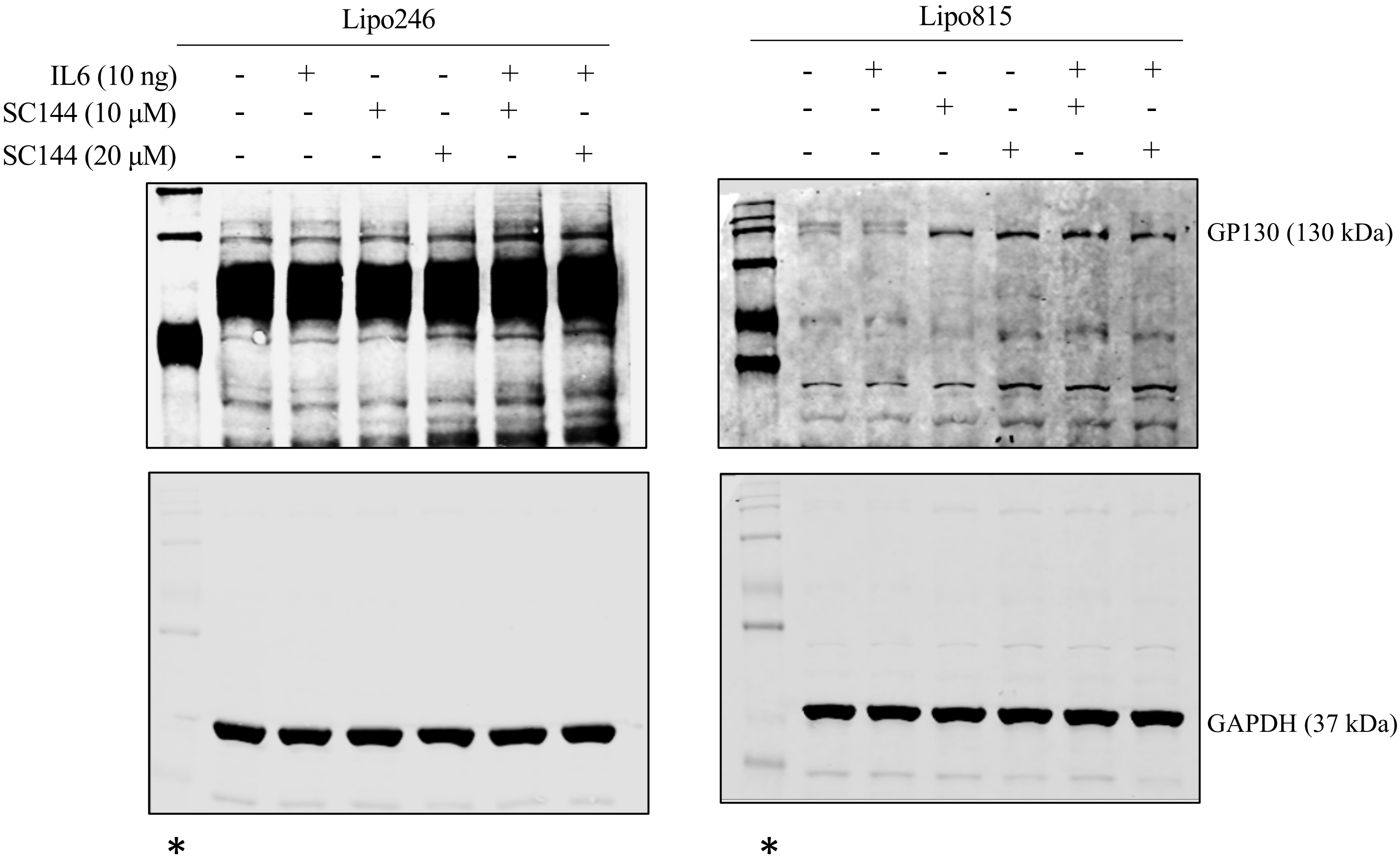

* First lane denotes ladder

Imaging and protein expression analyses were performed with the Odyssey CLx imager.

Fig 7B. Whole-cell STAT1 and STAT3 protein levels in Lipo815 cells treated with IL6 and SC144.

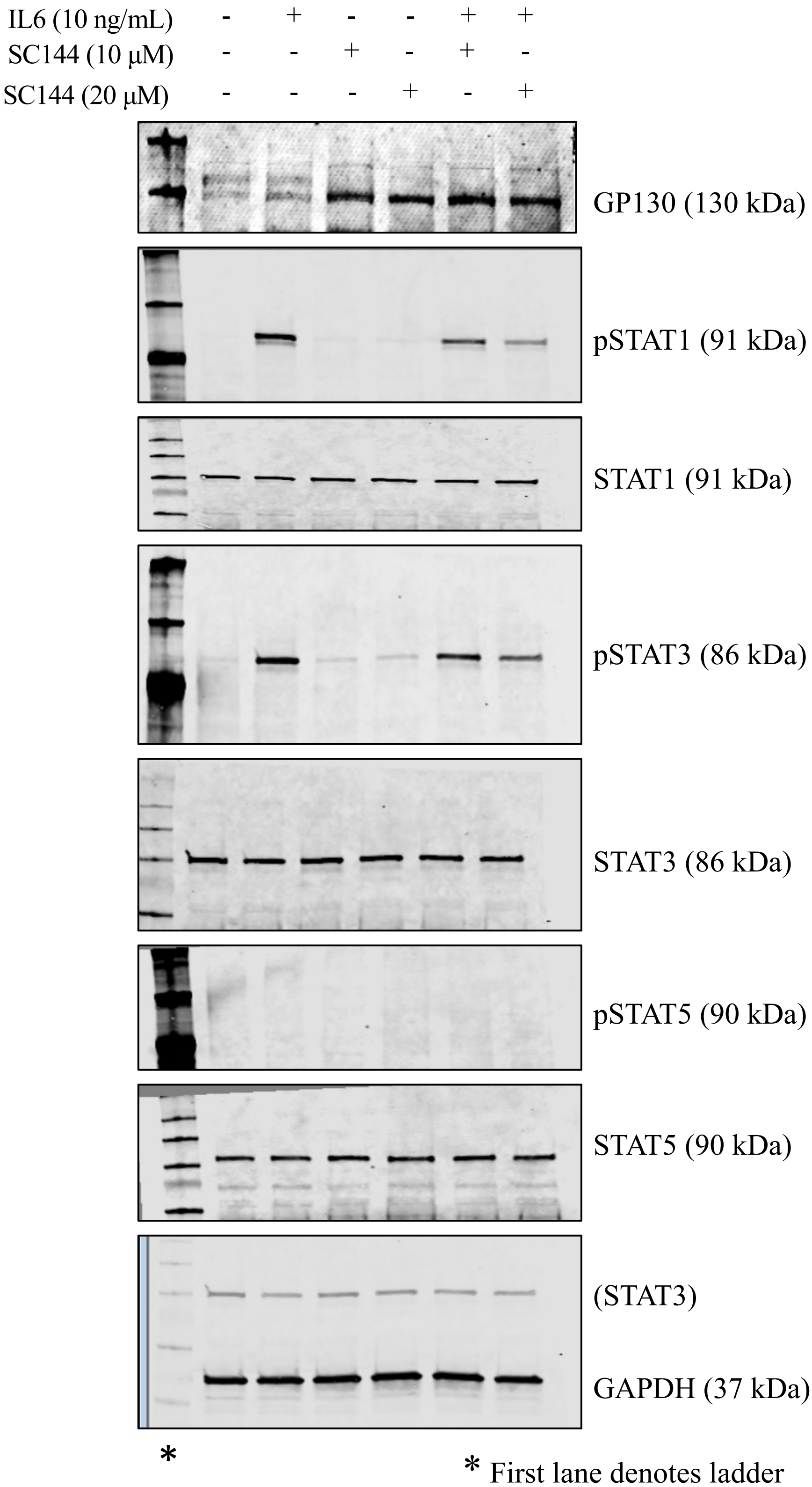

* First lane denotes ladder

Imaging and protein expression analyses were performed with the Odyssey CLx imager.

Fig 7C. Nuclear and cytoplasmic STAT1 and STAT3 protein levels in Lipo815 cells treated with IL6 and SC144.

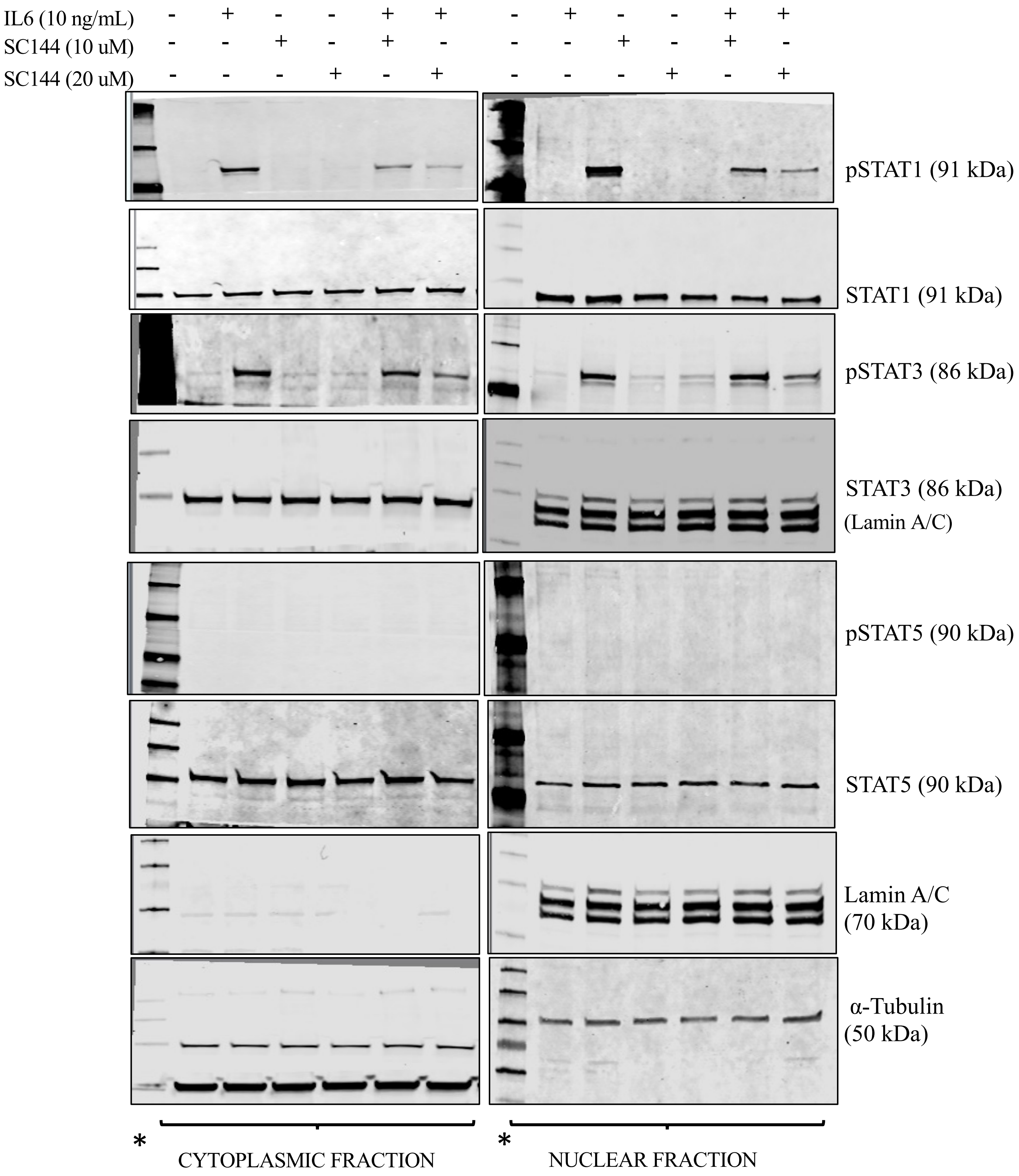

* First lane denotes ladder

Imaging and protein expression analyses were performed with the Odyssey CLx imager.

Fig 7D. GP130 and MDM2 expression in Lipo246 cells treated with IL6 and/or SC144

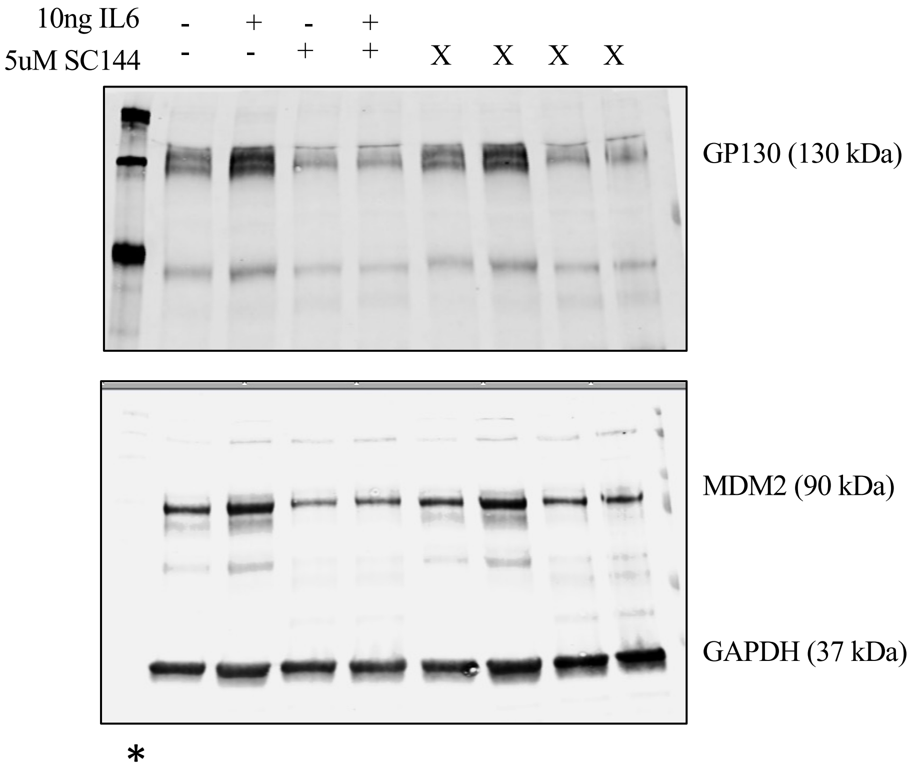

* First lane denotes ladder

Imaging and protein expression analyses were performed with the Odyssey CLx imager.

Fig 8B. MDM2-full length and MDM2-ALT1 Isoform Expression in DDLPS cells Treated with IL6 and/or SC144

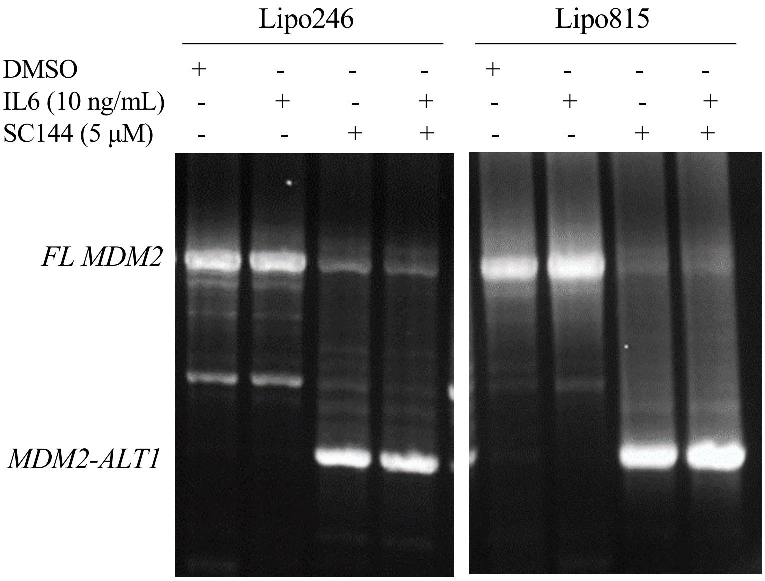

Imaging analyses were performed with a CCD imager.

Fig 8C. MDM2-full length and MDM2-ALT1 Isoform Expression in Lipo246 Xenograft Mice Treated with SC144

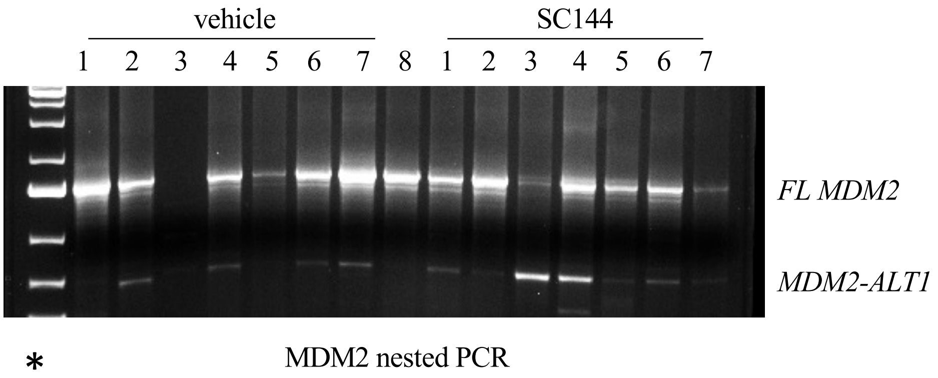

* First lane denotes ladder

Imaging analyses were performed with a CCD imager.

Supplemental Fig 1. ERK1/2 is constitutively active in DDLPS.

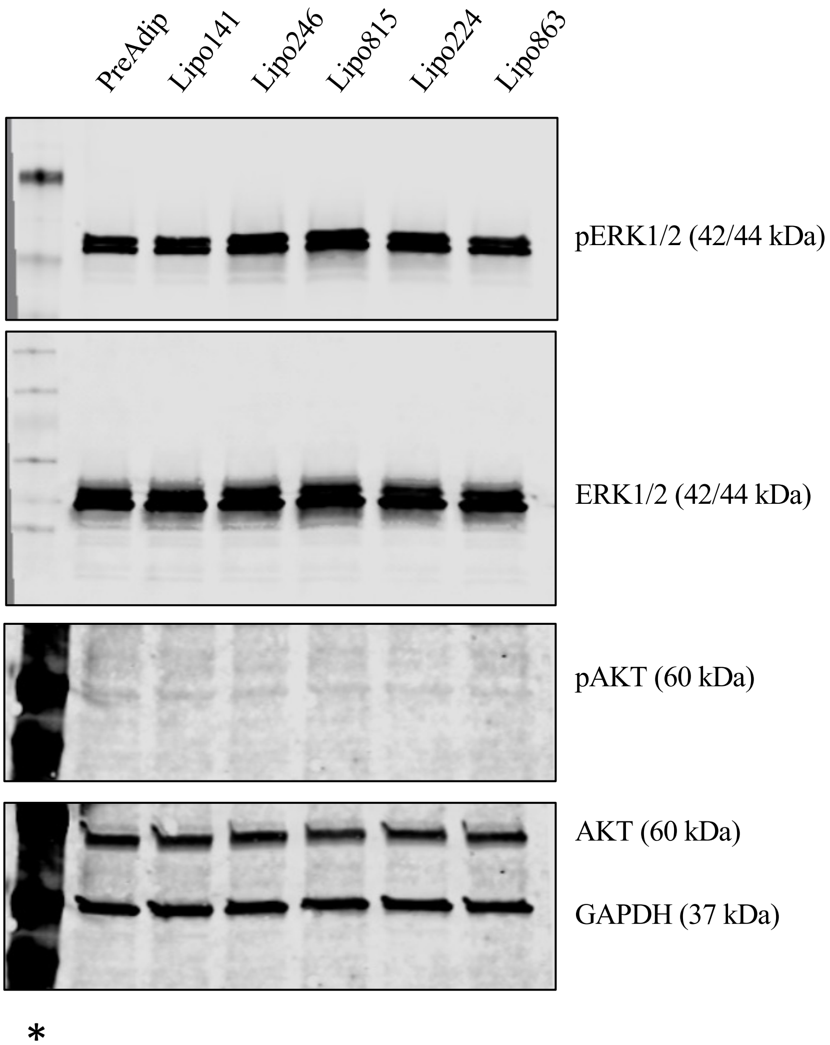

* First lane denotes ladder

Imaging and protein expression analyses were performed with the Odyssey CLx imager.

Supplemental Fig 2. The addition of IL6 to serum-starved DDLPS cells does not alter AKT activation or pERK1/2 expression.

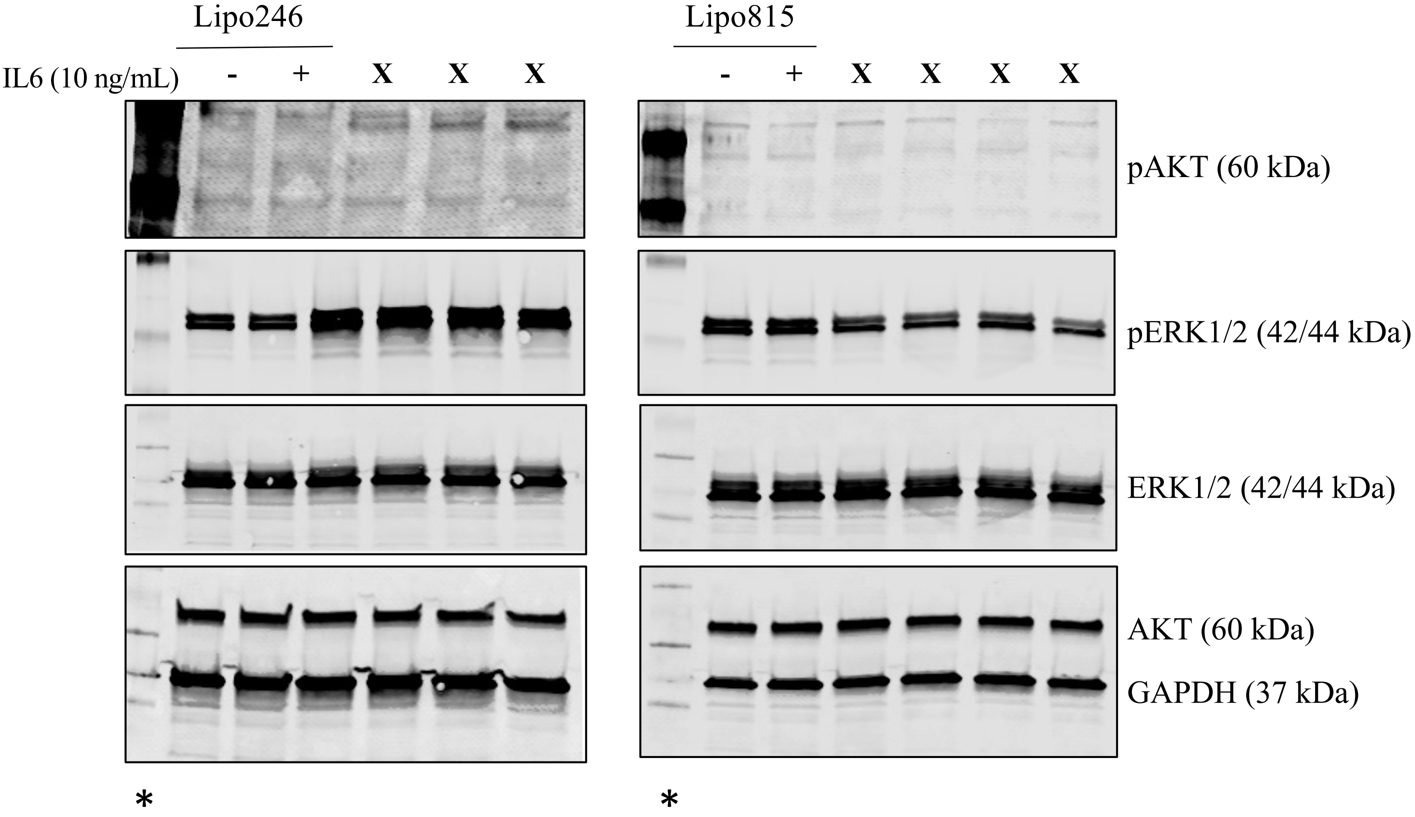

* First lane denotes ladder

Imaging and protein expression analyses were performed with the Odyssey CLx imager.

Supplemental Fig 4. MAB206 abrogates IL6-medicated STAT activation in Lipo815 cells

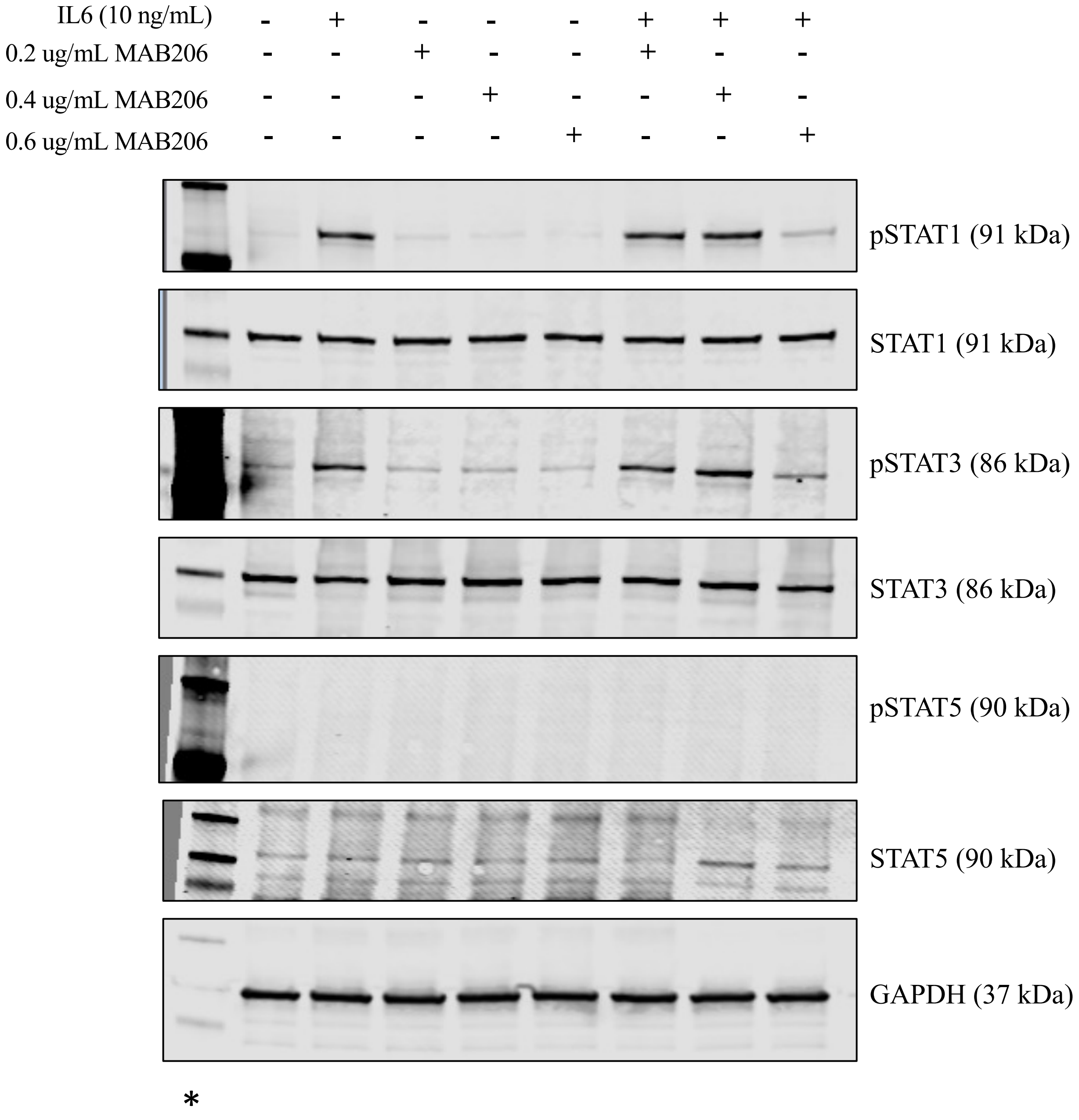

* First lane denotes ladder

Imaging and protein expression analyses were performed with the Odyssey CLx imager.

Supplemental Fig 6. GP130 knockdown decreases full-length *MDM2* mRNA expression and increases *MDM2-ALT1* production in Lipo246 cells

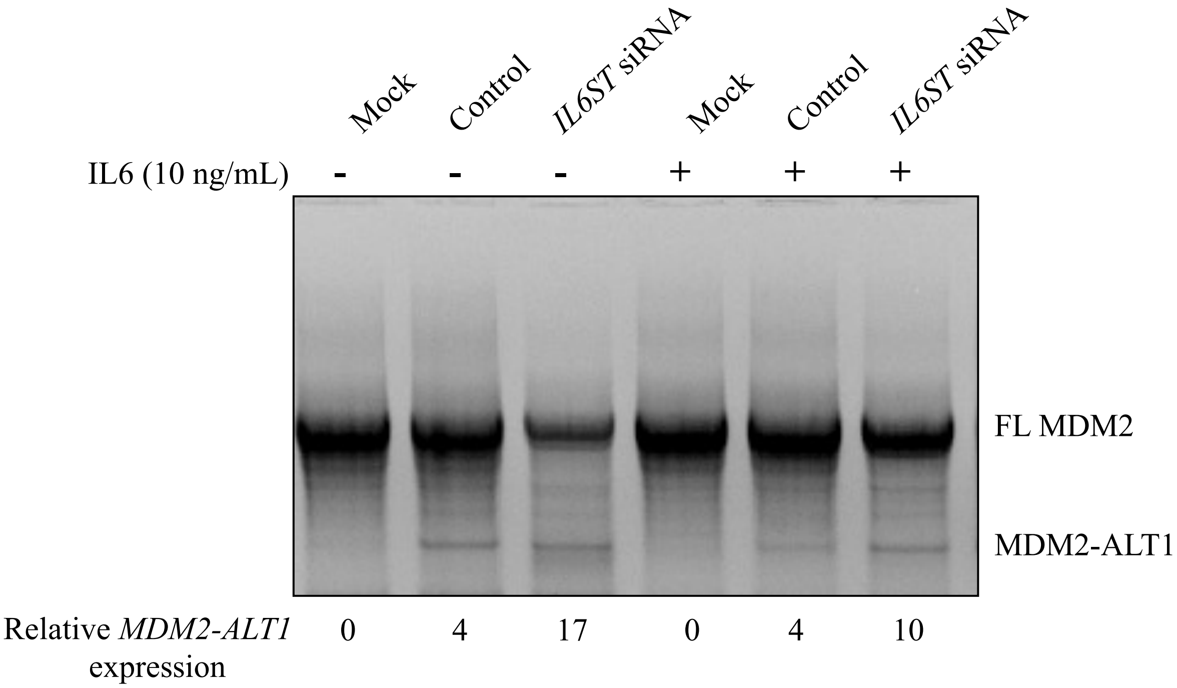

Imaging analyses were performed with a CCD imager.
